# The Specific Cleavage of Lactone Linkage to Open-Loop in Cyclic Lipopeptide during Negative ESI Tandem Mass Spectrometry: The Hydrogen Bond Interaction Effect of 4-Ethyl Guaiacol

**DOI:** 10.1371/journal.pone.0104835

**Published:** 2014-08-21

**Authors:** Mengzhe Guo, Youlu Pan, Rong Zhang, Yang Cao, Jianzhong Chen, Yuanjiang Pan

**Affiliations:** 1 Department of Chemistry, Zhejiang University, Hangzhou, China; 2 College of Pharmaceutical Sciences, Zhejiang University, Hangzhou, China; 3 School of Biotechnology, Jiangnan University, Wuxi, China; Xuzhou Medical college, China

## Abstract

Mass spectrometry is a valuable tool for the analysis and identification of chemical compounds, particularly proteins and peptides. Lichenysins G, the major cyclic lipopeptide of lichenysin, and the non-covalent complex of lichenysins G and 4-ethylguaiacol were investigated with negative ion ESI tandem mass spectrometry. The different fragmentation mechanisms for these compounds were investigated. Our study shows the 4-ethylguaiacol hydrogen bond with the carbonyl oxygen of the ester group in the loop of lichenysins G. With the help of this hydrogen bond interaction, the ring structure preferentially opens in lactone linkage rather than O-C bond of the ester-group to produce alcohol and ketene. Isothermal titration ^1^H-NMR analysis verified the hydrogen bond and determined the proportion of subject and ligand in the non-covalent complex to be 1∶1. Theoretical calculations also suggest that the addition of the ligand can affect the energy of the transition structures (TS) during loop opening.

## Introduction

Rapid developments and technological advances in the field of mass spectrometry have made electrospray ionization (ESI)-tandem mass spectrometry (MS/MS) a powerful tool for the analysis of peptides and proteins [1]–[2] with the capability to routinely identify sequences of tens of thousands of proteins [3]–[4]. Most analyses were performed using positive ion mode because the ionization of peptides and proteins in negative ion mode is relatively limited [5]. However experiments in negative ionization mode can also reveal important information in the investigation of peptide and protein structure and function [6]. Hydrophobic amide groups are typically positioned at the C-termini of peptides and are thought to be the key binding sites for biological activity. Negative ion mode can be used to more easily distinguish these amide groups as they are easily deprotonated [8]. Several works have been reported that utilized negative ion mode to study drugs, peptides, and proteins [7]–[8]. Cassady observed different fragmentation patterns in negative ion mode between nearly identical peptides which can provide insight into the structure of the peptides and improved identification of unknown peptides [9]. Moore utilized negative ion mode to investigate the fragmentation chemistry of anionic, hydrogen-deficient and radical peptides [10].

Microorganisms are known to produce peptides, especially cyclic peptides, with diverse biological activities that are of great interest to researchers in many fields. These peptides are produced from endophytic organisms, bacteria, and fungi [11]–[12]. The cyclic peptides of interest are formed via reactions with an ester group or a disulfide bond [13]–[14]. Studying the cleavage of cyclic peptides can help to elucidate their structure and function in microorganisms.

**Figure 1:**
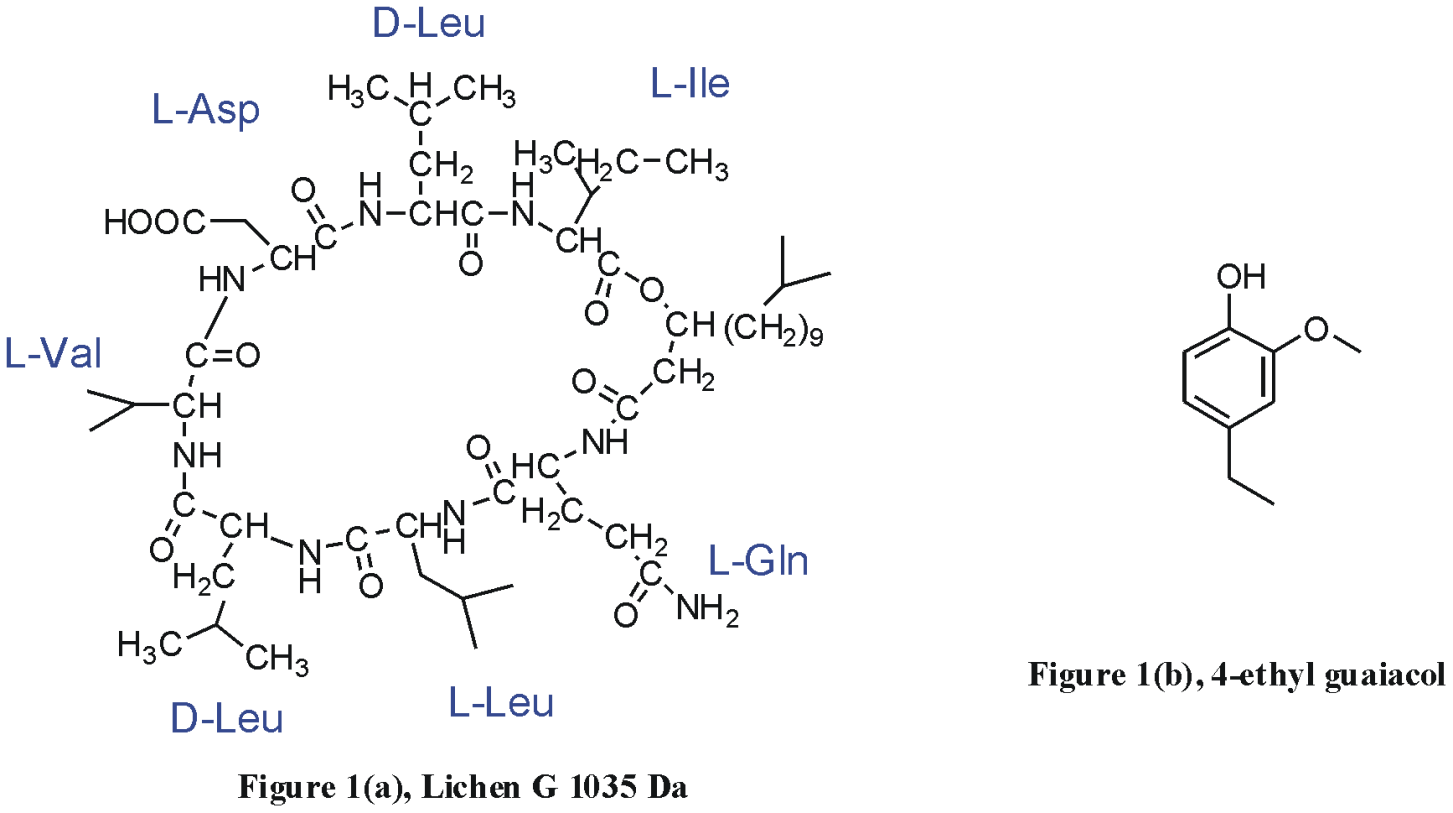
(a) lichensins G 1035 Da, (b) 4-ethyl guaiacol.

Surfactin is a bacterial cyclic lipopeptide produced by the Gram-positive, endospore-forming bacteria, *Bacillus subtilis*. It contains a heptapeptide with a hydrolysable ester linkage and a variable aliphatic chain of 13–15 carbon atoms. Because of its amphiphilic properties, which allow it to be stable in both hydrophilic and hydrophobic environments, surfactin is commonly used as an antibiotic. Previous studies reported that surfatin exhibits effective anti-tumour, anti-inflammatory and immunosuppressive activity [15]–[17]. The lichenysin and four homolog compounds produced by *Bacillus licheniformis* are cyclic lipopeptides in the surfactin family. The primary homolog, lichenysins G (MW 1035 Da), contains the previously mentioned heptapeptide and an aliphatic chain of 15 carbon atoms, which is shown in Figure 1a.

Mass spectrometry is a powerful technique for the study of cyclic lipopeptide structure and function. Pathak used HPLC coupled to ESI-MS to identify several new fengcin variants and also found four major sites of heterogeneity between them [18]. Samgina utilized collision-induced dissociation (CID) to elucidate the sequences of natural, non-tryptic peptides with C-terminal disulfide cycle [19]. Using mass spectrometry to study non-covalent complexes of peptides and ligands can reveal peptide cleavage sites. O'Hair used mass spectrometry to investigate the gas phase chemistry of proton bound oligosaccharide (S) –ligand (L) non-covalent complexes and found that a ladder series is produced by ligand induced oligosaccharide bond cleavage [20].

In this work, lichenysins G, the predominant lichenysin homolog, was selected as the model to investigate the fragmentation mechanism using negative ion tandem mass spectrometry. 4-ethylguaiacol, an important pharmaceutical intermediate, was added as the ligand and the structure is shown in Figure 1b. By comparing the different fragmentation of the lichenysins G and the non-covalent complex of lichenysins G and 4-ethylguaiacol, the detail of the interaction between this two compounds and the effects on fragmentation of lichenysins G induced by the ligand are expounded. The existing work [21]–[23] helping to propose the fragmentation mechanism and theoretical calculations were used to support the proposed mechanism.

## Experimental

### Materials

The four lichenysin targets (purity >90% by MS analysis) were provided by the School of Biotechnology, Jiangnan University. 4-ethylguaiacol (purity >95% by LC-MS analysis) was purchased from Sigma-Aldrich.

### Mass Spectrometry

All CID experiments were performed on a Bruker AmaZon ETD mass spectrometer (Bruker-Franzen Analytik GmbH, Bremen, Germany) equipped with a nanospray ionization source and an ion trap mass analyzer using negative ion mode. Nitrogen was used as nebulizing gas at a pressure of 10 psi and drying gas at a flow rate of 5 L·min^−1^. The drying gas temperature was 250°C and the capillary voltage was 4000 V. Samples were dissolved in acetonitrile/1% salicylic acid solution 99.9/0.1 (v:v) to form a 1×10^−8^ mol·L^−1^ and infused to the mass spectrometer with a syringe pump at a flow rate of 3 uL·min^−1^. The CID mass spectra were obtained with helium as the collision gas at suitable collision energy after isolation of the desired precursor ion. The mass window for precursor ion selection was between 0.8 and 1.0 *m/z* to ensure ^13^C isotopic ions were excluded. Data were acquired using the software Esquire 5.0 (Bruker).

### NMR Analysis

^1^H NMR and 2D NOESY experiments were carried out using a Bruker AMX 500 MHz instrument at 298 K with samples in d-DMSO. Typical parameters for ^1^H NMR experiments consisted of spectral width of 10 ppm, number of scans at 16 and relaxation delay at 1 s. 50–60° pulses with overall delay of 3 s between pulses were used. The time-domain data were exponentially multiplied before Fourier transformation with 0.0–0.5 Hz line broadening functions depending on the resulting S/N ratio of the spectrum. Tetramethylsilane (TMS) was used as internal reference for all spectra (0 ppm). For NOESY spectrum, the data was acquired with a mixing time of 60 ms and a relaxation delay at 2 s [24].

### Theoretical Calculations

The software program Amber was used to perform the conformational search of the non-covalent complex ion. Using molecular dynamics simulation of 100 ns, we divided the series of conformers into twenty groups and obtained twenty representative conformations. The global minimum energy of conformation was chosen by comparing all molecular dynamics simulation energy.

Potential energy surfaces (PES) were also used to take into consideration the deprotonated lichenysins G and non-covalent complex. The candidate structures of the reactants, products, intermediates and transition states were optimized by calculating the force constants. Calculations were performed to modify the initial structures with a deprotonated carboxyl group and compare the 1, 3 hydrogen transition structure (TS) energy from either the alpha carbon or aliphatic carbon to the oxygen in the ester group. All theoretical calculations were carried out by using the ONIOM method at the B3LYP/6−311++G (d,p) level of theory and PM6 theory in the Gaussian 03 program. No symmetry constrains were imposed on the optimizations. All optimized structures were subjected to vibrational frequency analysis for zero-point energy (ZPE) correction. The sum of electronic and thermal energies of the optimized structures was discussed.

## Results and Discussion

### Fragmentation of Lichenysins G and Non-covalent Complex

**Figure 2:**
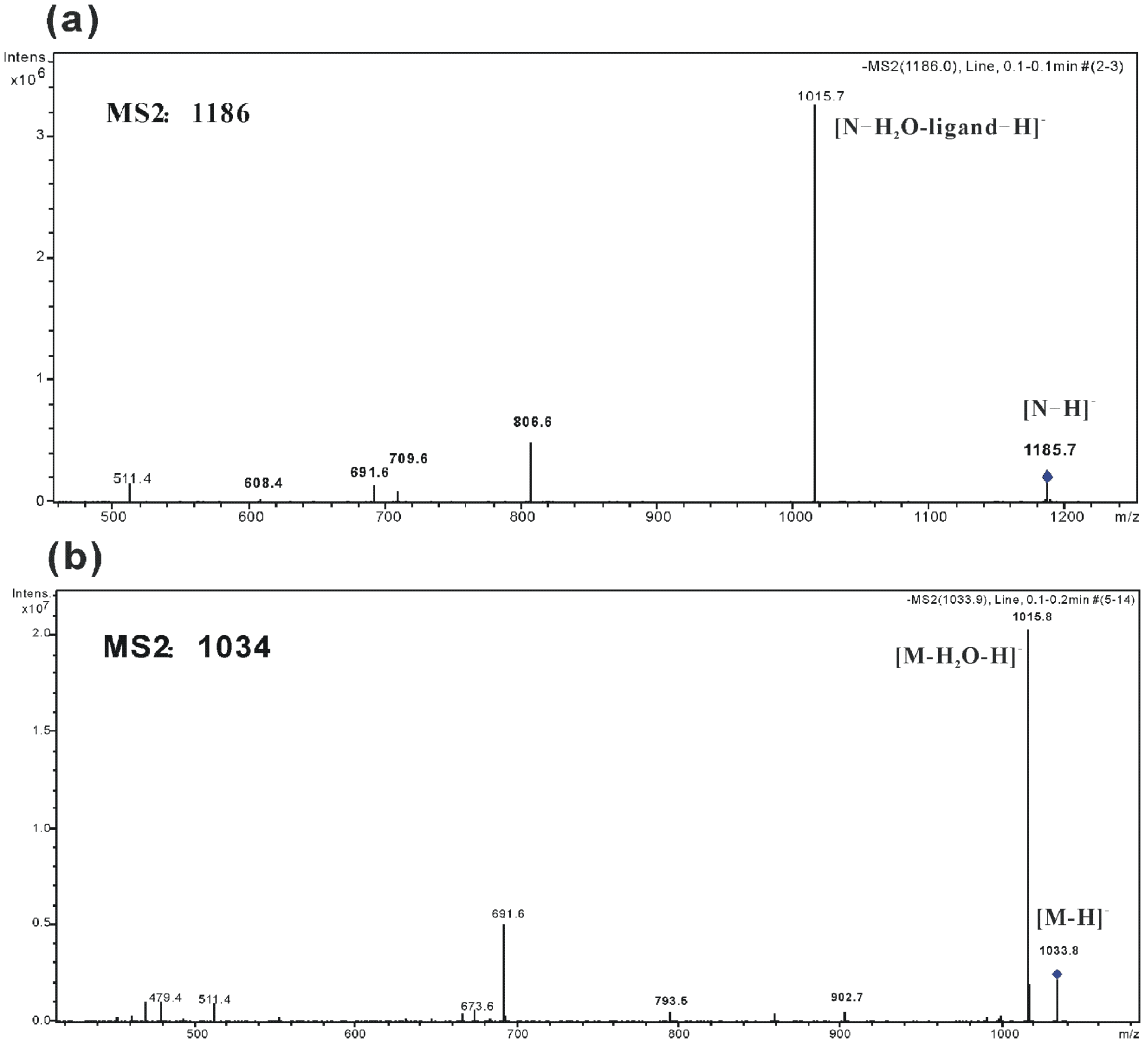
The fragmentation of non-covalent complex (a) and lichenysins G (b).

**Figure 3:**
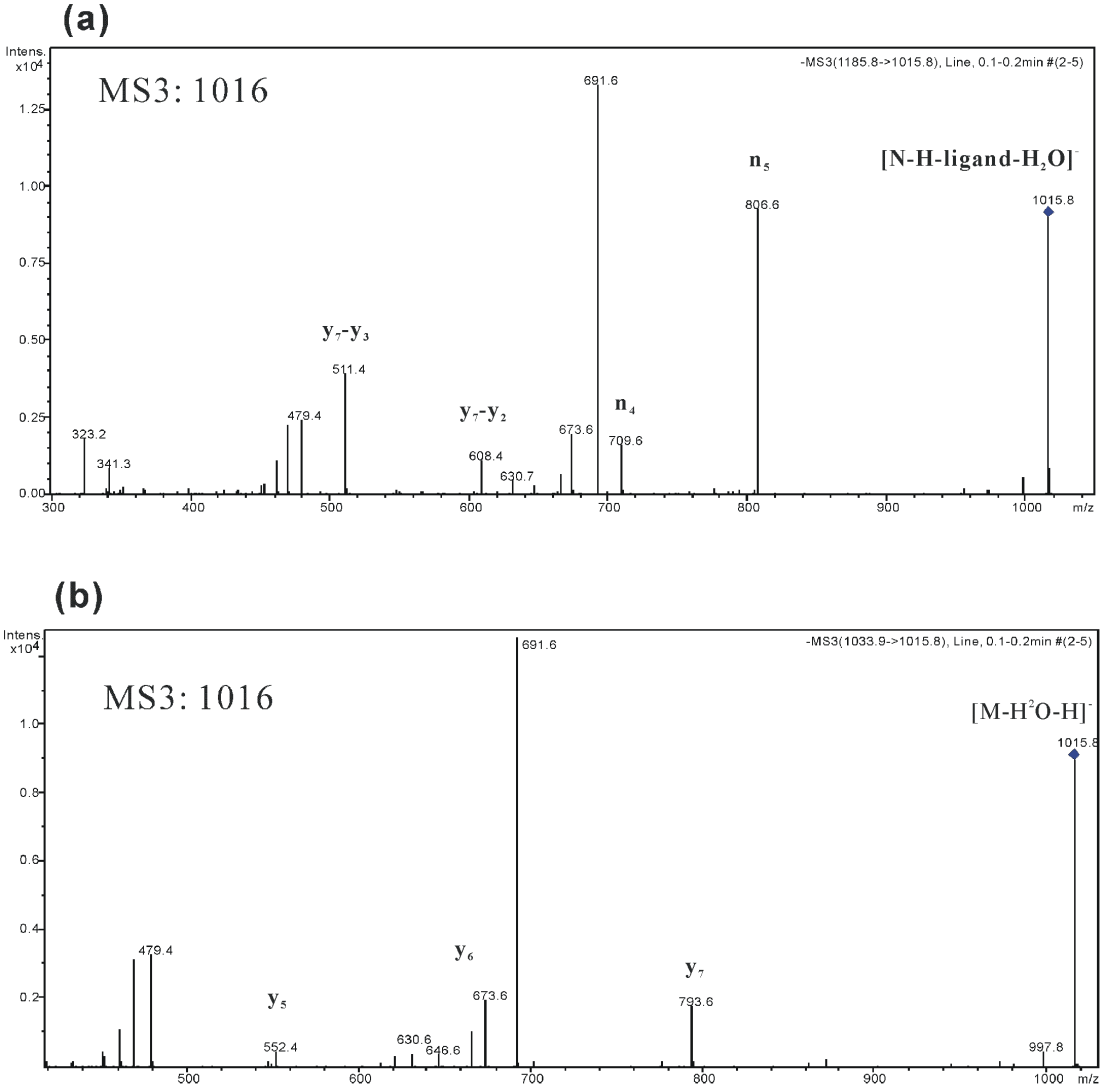
The MS^3^ fragmentation of 1016 Th ions of non-covalent complex (a) and lichenysins G (b).

The parent ion shown in Figure 2(a) is the non-covalent 1∶1 lichenysins G: 4-ethylguaiacol complex, [N-H]^−^ (*m/z* 1186), and four characteristic fragments for the complex were observed: *m/z* 1016, 807, 710 and 692. And the Figure 2(b) exhibits the fragmentation of lichenysins G in negative ion mode. The fragmentation of the lichenysins G anion, [M-H]^−^ (*m/z* 1034), resulted in three characteristic framents: *m/z* 1016, 794 and 692. In Table 1 which summarizes the primary fragments of M and N we can find the two targets have identical *m/z* values: *m/z* 1016 and *m/z* 692. Performing MS^3^ of *m/z* 1016 and *m/z* 692 can help determining whether these fragments with same molecular weight have the same molecular stucture. The results of MS^3^ are shown in Figure 3, Figure S1 in File S1 and Table 1. Figure 3(a) shows the MS^3^ fragmentation of *m/z* 1016 fragment from the non-covalent complex and Figure 3(b) shows the MS^3^ fragmentation of *m/z* 1016 fragment from lichenysins G, respectively. The primary MS^3^ fragments of 1016 for M are *m/z* 794 and 692, whereas the primary MS^3^ fragments of 1016 for N are *m/z* 807, 710 and 692. The different characteristic MS^3^ fragmentation patterns observed for M and N suggest that different molecular structures comprise the *m/z* 1016 MS^2^ fragment for each analyte. One the other hand, the MS^3^ fragmentaiton of *m/z* 692 fragments from N (Figure S1(a) in File S1) and M (Figure S1(b) in File S1) indicate that they have the same molecular structure because of their same fragmentations.

**Table 1:** Main ions of every fragmentation of Cyclic Lipopetide and Non-covalent complex.

	MS^2^				MS^3^				MS^3^			
Targets	I		II		I		II		I		II	
Parent ions	1034		1186		1016		1016		692		692	
	Ions	Abundance	Ions	Abundance	Ions	Abundance	Ions	Abundance	Ions	Abundance	Ions	Abundance
	1016	97.0%	1016	98.0%	794	16.7%	807	65.5%	674	6.8%	674	46.0%
	903	4.8%	807	16.5%	692	100%	710	10.5%	631	6.5%	647	6.3%
	794	4.9%	710	3.5%	674	17.5%	692	98.0%	621	3.2%	631	8.1%
	692	28.0%	692	6.5%	522	5.0%	608	7.3%	480	95.0%	621	3.1%
	675	4.9%	608	0.5%	479	26.7%	511	29.0%	470	64.5%	480	83.0%
	511	9.7%	511	6.5%	…	…	479	18.0%	…	…	470	75.0%
	…	…	…	…			…	…			…	…

I fragmentation of Cyclic Lipopetide.

II fragmentation of Non-covalent complex.

### Fragmentation Mechanisms of Lichenysins G and Non-covalent Complex

Further analysis of the MS^3^ fragmentation of these two *m/z* 1016 fragment ions from lichenysins G and non-covalent complex, can help elucidate the fragmentation mechanisms for each analyte. The specific cyclic structure of lichenysins G should be taken into consideration; therefore, the open loop of lichenysins G is hypothesized as the initial process of fragmentation.

**Figure 4:**
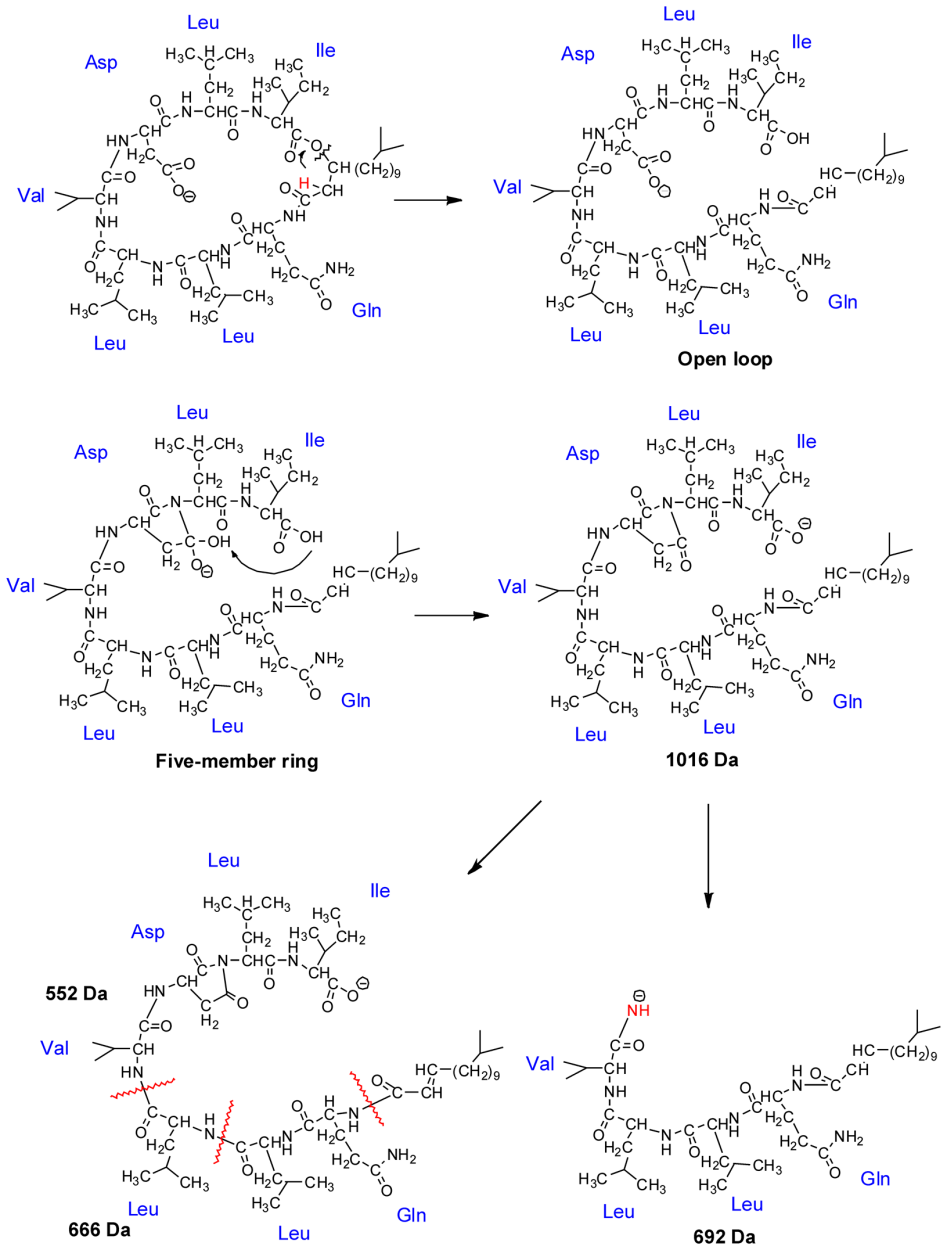
The fragmentation mechanisms of lichenysins G.

Figure 4 shows the pathways rationalizing the fragmentation of M in negative ion mode. At the beginning, the carboxyl of the Asp side chain loses a hydrogen forming the [M-H]^−^ ion. Next the hydrogen from the methylene in the middle of aliphatic chain and carbonyl (colored in red), transfers to the oxygen of ester group via 1,3 hydrogen migration. Thereafter, the carbon (from aliphatic chain) - oxygen (from ester group) bond is broken, sequentially opening the loop and forming a carboxyl and ethylene at both ends respectively [24]–[28]. Finally, the compound, which loses water after ring opening, forms the [M-H-H_2_O]^−^ (*m/z* 1016). The loss of water may have two possible pathways. One pathway could be that the tertiary amine of Leu attacks the carbon of the adjacent Asp carboxyl group to form a five-member ring. The hydrogen of the carboxyl group formed by the ring opening can transfer to the hydroxyl formed by the five-member ring as mobile proton, resulting in the water loss [29]–[33]. The second potential pathway is that the carboxyl formed by the ring opening can directly lose water and form the ketene. The first pathway is more probable and supports in the following pages.

Both MS^2^ of [M-H]^−^ and MS^3^ of [M-H-H_2_O]^−^ generate the fragment *m/z* 794, shown in Figure 2 and 3. The fragment ion *m/z* 794 is formed from the open ring structure of [M-H-H_2_O]^−^. The ring opening results in linear precursor ions, which undergo subsequent cleavages as shown in figure 4, and then creates a y_7_ ion which generated the *m/z* 794 by the neutral loss of an unsaturated ketene group. In addition, this fragmentation may continue and produce the y_6_ (*m/z* 666) and y_5_ (*m/z* 552) fragmentation ions by losing Gln and Leu respectively. The three fragments listed above can demonstrate that the ring is indeed open in the location of O-C bond between the ester group and aliphatic chain.

Figure 4 also illustrates the mechanism pathway that rationalizes the neutral loss of *m/z* 342 from lichenysins G to form the *m/z* 692 ion. This process proceeds by the N-C bond dissociating from the amine and alpha carbon of the Asp side chain and produces the neutral loss of a tripeptide containing Asp, Leu and Ile. Moreover, the cleavage of the N-C bond rationalizes the mechanism of water loss described above, because the stable five-member ring facilitates the N-C bond dissociation.

**Figure 5:**
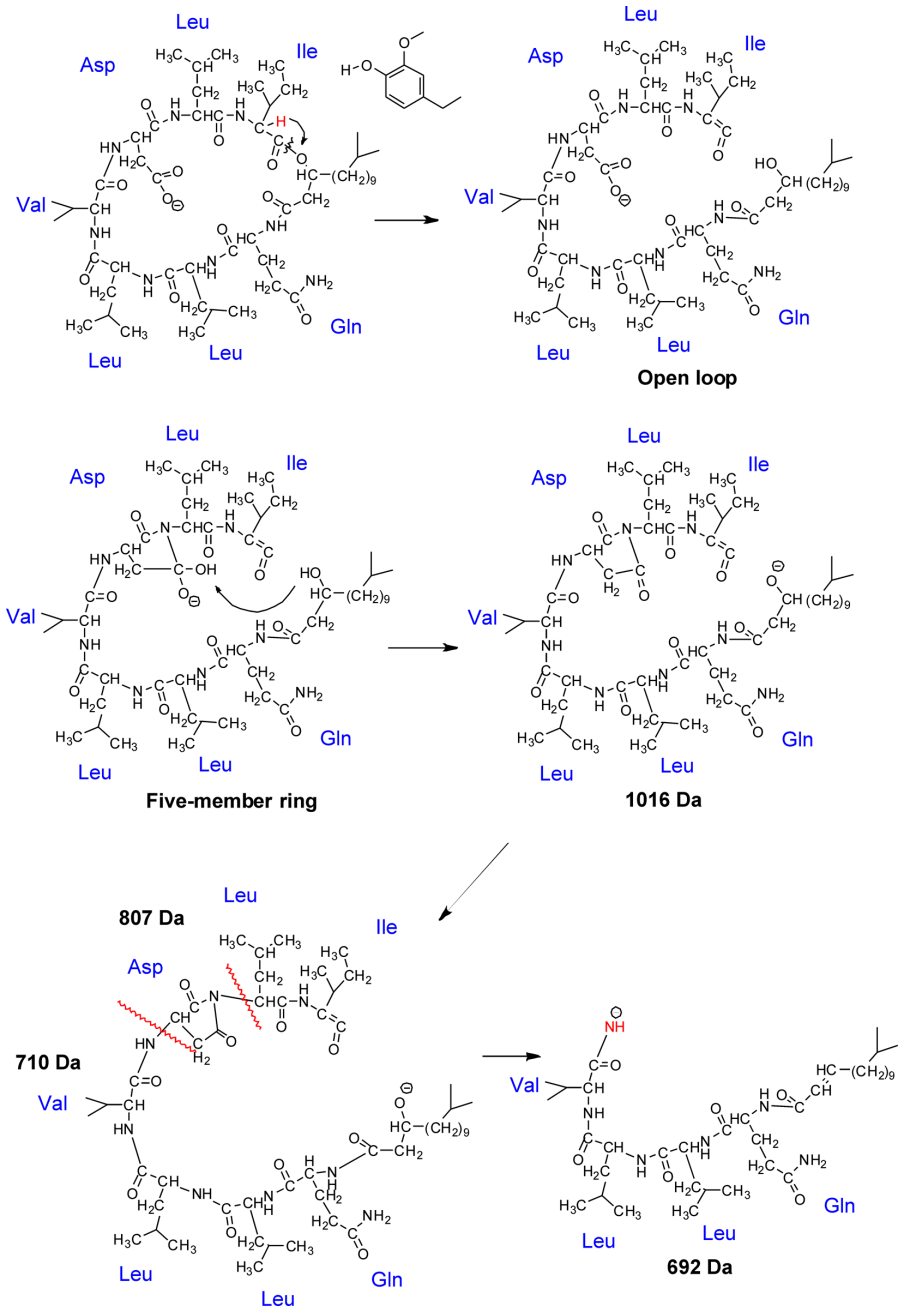
The fragmentation mechanisms of non-covalent complex.

Compared to figure 4 there is prominent difference in the fragmentation mechanisms of [N-H]^−^ in the way of open loop. It is suggests that the ligand has no direct participation in the fragmention of N from Figure 2 and 3 by means of determining all the fragmentations belong to the subject M. Figure 5 illustrates the pathway for formation of fragmentation ion *m/z* 1016. First the non-covalent complex may lose the neutral micromolecule ligand and next the hydrogen from alpha carbon of Ile transfers to the oxygen of ester group via 1,3 hydrogen migration. A cleavage occurs inside the lactone linkage forming a ketene and an alcohol. Finally the loop opening results in a loss of water and the formation of [N-H-ligand-H_2_O]^−^ (*m/z* 1016). The water loss also proceeds by the nucleophilic attack of the Asp carboxyl carbon on the amidogen from the adjacent Leu, forming the five-member ring. The hydrogen of the alcohol formed during ring opening transfers to the hydroxyl formed by the five member ring as mobile proton resulting in the water loss.

**Figure 6:**
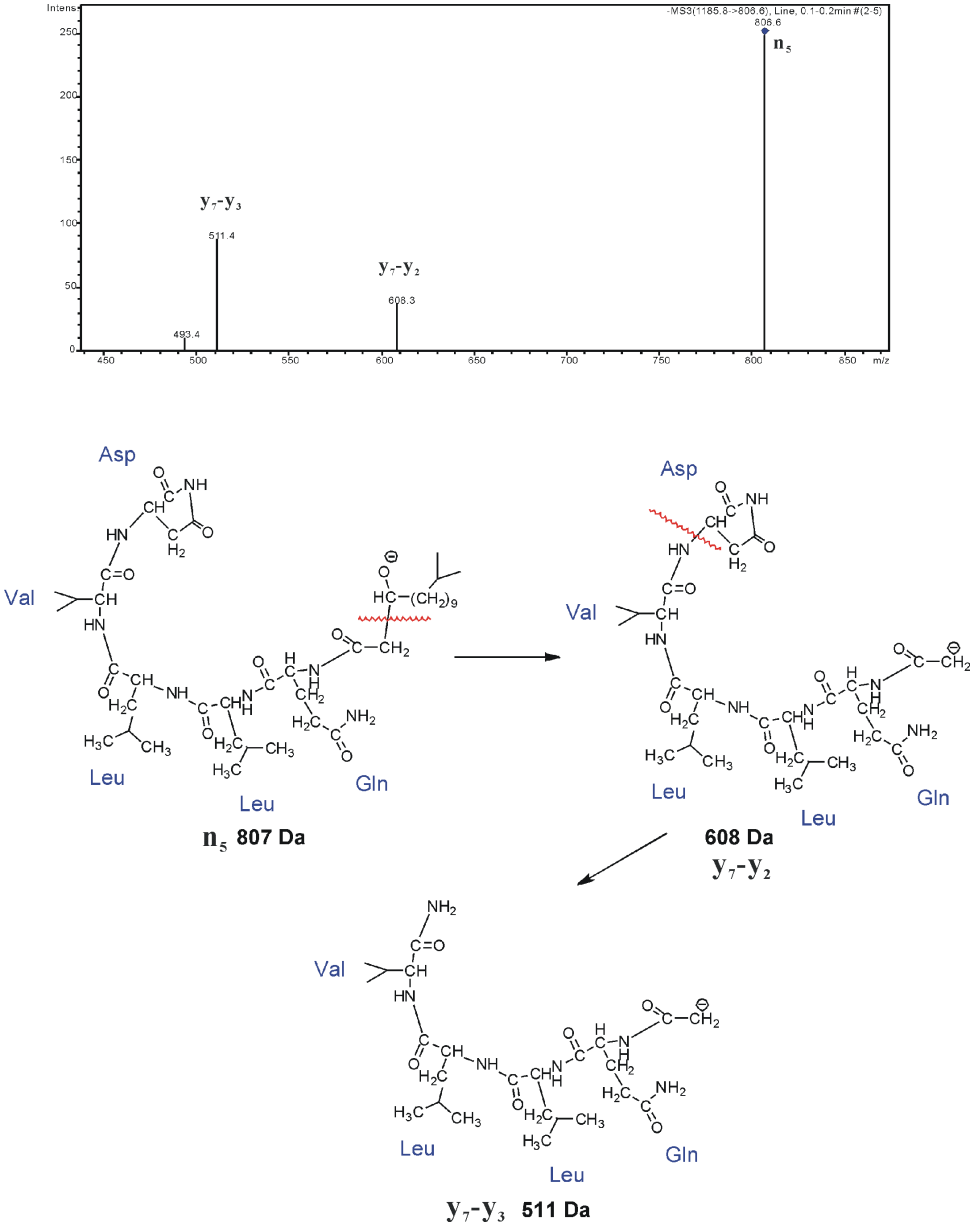
The MS^3^ analysis and fragmentation mechanisms of n_5_ (m/z 807).

The ions with *m/z* 807 and 710 are the fragmentation products of linear precursor ions formed by ring opening. They also have a relationship with the formation of the five-member ring in Asp. When the N-C bond from the amine and alpha carbon of Leu is cleaved, the neutral dipeptide, containing Leu and Ile, may be lost forming the n_5_ ion with *m/z* 807. If the five-member ring in the Asp side chain is lost as well, the remaining product is the n_4_ ion with *m/z* 710. The n_4_ ion can easily lose water from the deprotonated alcohol in the terminal of aliphatic chain and form the ion with *m/z* 692 which is a fragment of [M-H]^−^ shown above. The fragmentation mechanism for forming n_5_ is also supported by the MS^3^ results of the n_5_, which is shown in Figure 6. In this fragmentation the cleavage of the C-C bond in the linear aliphatic chain to lose the aliphatic ketone produce the the ion [y_7_-y_2_] (*m/z* 608) and continue losing the five-member ring of the Asp side chain to produce the ion [y_7_-y_3_+NH_2_] (*m/z* 511). These two ions are also found in the MS^3^ results of [N-H-ligand-H_2_O]^−^ and support the structure of the n_5_ ion. In addition, the structure of n_5_ also can show the fragmentation mechanism of [N-H]^−^ and the location of the loop opening.

Through the comparative analysis of the different fragmentation mechanisms of lichenysins G and the non-covalent complex, we infer that the ring opening location of lichenysins G can exchange from oxygen and carbon in the aliphatic bond to the carbonyl and oxygen bond of the ester group caused by the effect of the micromolecule ligand. The linear product ion transfers from the carboxyl and ethylene to the ketene and alcohol. In addition, the effective interaction of the micromolecule ligand may be the hydrogen bond between the phenolic hydroxyl group in the ligand and the oxygen of ester group carbonyl in the lichenysins G.

### ^1^H-NMR Analysis

**Figure 7:**
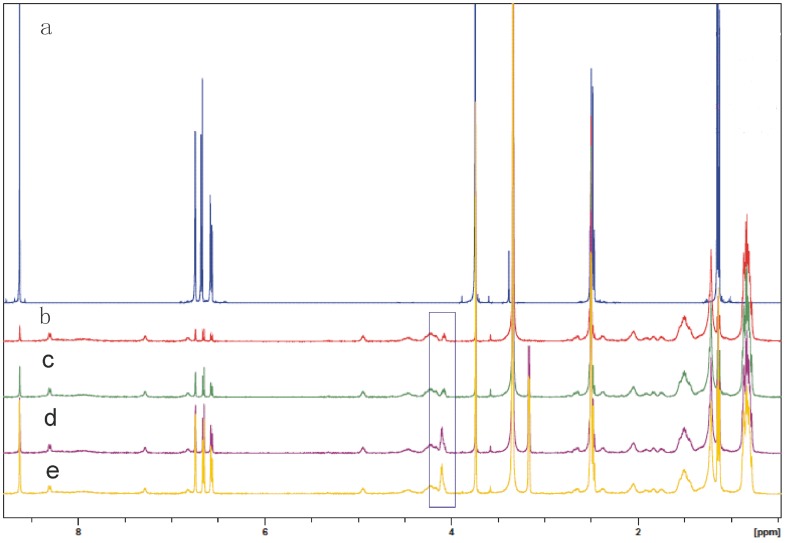
^1^H NMR spectra (400 MHz) of non-covalent complex lichenysins G:4-Ethyl Guaiacol in DMSO at 25°C; b) 4∶1; c) 2∶1; d) 1∶1; e) 1∶2.

To verify the ESI-MS result of the non-covalent complex, the types of interactions between lichenysins G and 4-ethylguaiacol were determined by ^1^H-NMR. The ^1^H-NMR spectroscopy of lichenysins G was in accordance with the literature [34]. Spectroscopic titration suggested that lichenysins G and 4-ethylguaiacol could form stable 1∶1 complex. The _α_H on alpha-carbon of Ile (δ = 4.12 ppm) had a remarkable shift and change in peak shape. Additional evidence of such interaction was provided by 2D ^1^H-^1^H NOESY NMR spectroscopy. The NOE correlations (_α_H, H_i_) between the H on alpha-carbon of Ile and the hydroxyl of 4-ethylguaiacol confirm the interaction (Figure 7, Figure S2 in File S1).

### Theoretical Calculations

**Figure 8:**
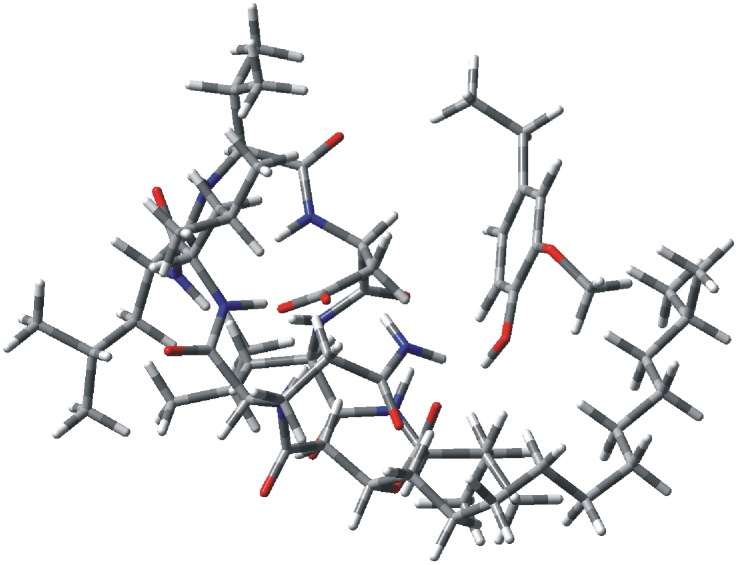
The dynamic conformation of lichenysins G and ligand.

The crystal structure of surfactin was used as the reference when performing the theoretical calculations of the lichenysins G, because the amino acid sequence and cis-trans structure of lichenysins G are similar to surfactin. Molecular dynamics simulations of the hydrogen bond interaction between lichenysins G and the ligand provided a steady dynamic conformation (Figure 8).

**Figure 9:**
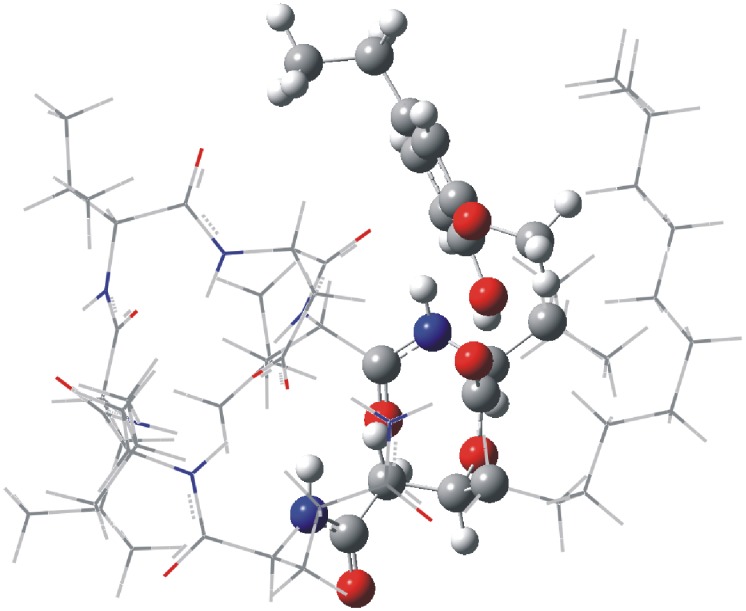
The mixed level of theory in which the nucleus calculated at B3LYP/6−31+G(d) is shown as ball & bond type and periphery region calculated at PM6 is shown as wireframe.

The mechanisms were further explored via theoretical calculations at an ONIOM level of theory. Figure 9 shows that the ligand and the ester group where hydrogen transfer and loop opening occur were approached as the nucleus and used RB3LYP/6−311++G(d,p) level of theory. The rest of the peptide was treated as a periphery region and treated with PM6 level of theory. When the ligand did not participate, the individual lichenysins G was optimized as the potential energy zero. The transition state energy of 1,3 hydrogen migration of the methylene hydrogen in the middle of aliphatic chain carbonyl to the oxygen of ester group (TS1-1) is 56.09 kcal/mol, 25.53 kcal/mol less than the transition state energy of 1,3 hydrogen migration of the alpha-carbon hydrogen of Ile to the oxygen of the ester group (TS1-2, 81.62 kcal/mol) (Table S1 in File S1). This result confirms the fragmentation mechanism of ring opening without the ligand. However, with the hydrogen bond interaction of the ligand, the transition state energy of 1,3 hydrogen migration of the alpha-carbon hydrogen of Ile to the oxygen of the ester group (TS2-2) prominently decreases to 76.76 kcal/mol but the transition state energy of 1,3 hydrogen migration of the methylene hydrogen in the middle of aliphatic chain carbonyl to the oxygen of ester group (TS2-1) was not founded. We observed that the conformation of TS2-1 changed back to the reactant structure when calculating the transfer structure. This could potentially be because the hydrogen bond interaction stabilizes the C-O bond of TS2-1 and makes the C-O bond of TS2-2 easier to cleave. Therefore this result supports the open-loop mechanism of the non-covalent complex.

## Conclusion

In this work we found a difference in fragmentation mechanisms of lichenysins G when the ligand, which contains phenol hydroxyl group, was present compared to the direct dissociation of lichenysins G alone using ESI tandem mass spectrometry. The hydrogen bond between phenol hydroxyl of the ligand and the carbonyl oxygen of the ester group in the cyclic lipopeptide ring, which was confirmed by NMR, can promote cleavage of the C-O bond of the ester group, resulting in loop opening and ketene and alcohol generation. However with the absence of the hydrogen bond effect, the location of the loop opening of cyclic lipopeptide changes to the O-C bond beside the ester group, producing ethane and a carboxyl as reported in the literature. Molecular dynamics simulation supplies further evidence that there is a stabilized hydrogen bond and the potential energy surface calculations for the hydrogen translation structures energy also support the fragmentation mechanism of the non-covalent complex. The results of this work can provide the theoretical support for the research of metabolites with diverse biological activities produced from endophytic organisms.

### Electronic Supplementary Material

The online version of this article contains supplementary material, which is available to authorized users.

## Supporting Information

File S1 **Supporting figures and table.** Figure S1. The MS^3^ fragmentation of 1016 Da ions of non-covalent complex (a) and lichenysins G (b). Figure S2. ^1^H-^1^H NOESY spectra (400 MHz) of non-covalent complex (1∶1 in molar ration)in DMSO. Table S1, The calculation of non-covalent complex and lichenysins G with ONIOM level of theory.
(DOC)
