# Supplementary material for: The Specific Cleavage of Lactone Linkage to Open-Loop in Cyclic Lipopeptide during Negative ESI Tandem Mass Spectrometry: The Hydrogen Bond Interaction Effect of 4-Ethyl Guaiacol
Source: PLoS One. 2014 Aug 21;9(8):e104835. doi: 10.1371/journal.pone.0104835 (PMC4140680; doi:10.1371/journal.pone.0104835)
Supplement: File S1 — Supporting figures and table. Figure S1. The MS3 fragmentation of 1016 Da ions of non-covalent complex (a) and lichenysins G (b). Figure S2. 1H-1H NOESY spectra (400 MHz) of non-covalent complex (1∶1 in molar ration)in DMSO. Table S1, The calculation of non-covalent complex and lichenysins G with ONIOM level of theory. (DOC) [file pone.0104835.s001.doc]

*Supporting Information*

Figure S1. The MS^3^ fragmentation of 1016 Da ions of non-covalent complex (a) and lichenysins G (b).

Figure S2. ^1^H-^1^H NOESY spectra (400MHz) of non-covalent complex (1:1 in molar ration )in DMSO.

Table S1, The calculation of non-covalent complex and lichenysins G with ONIOM level of theory

*
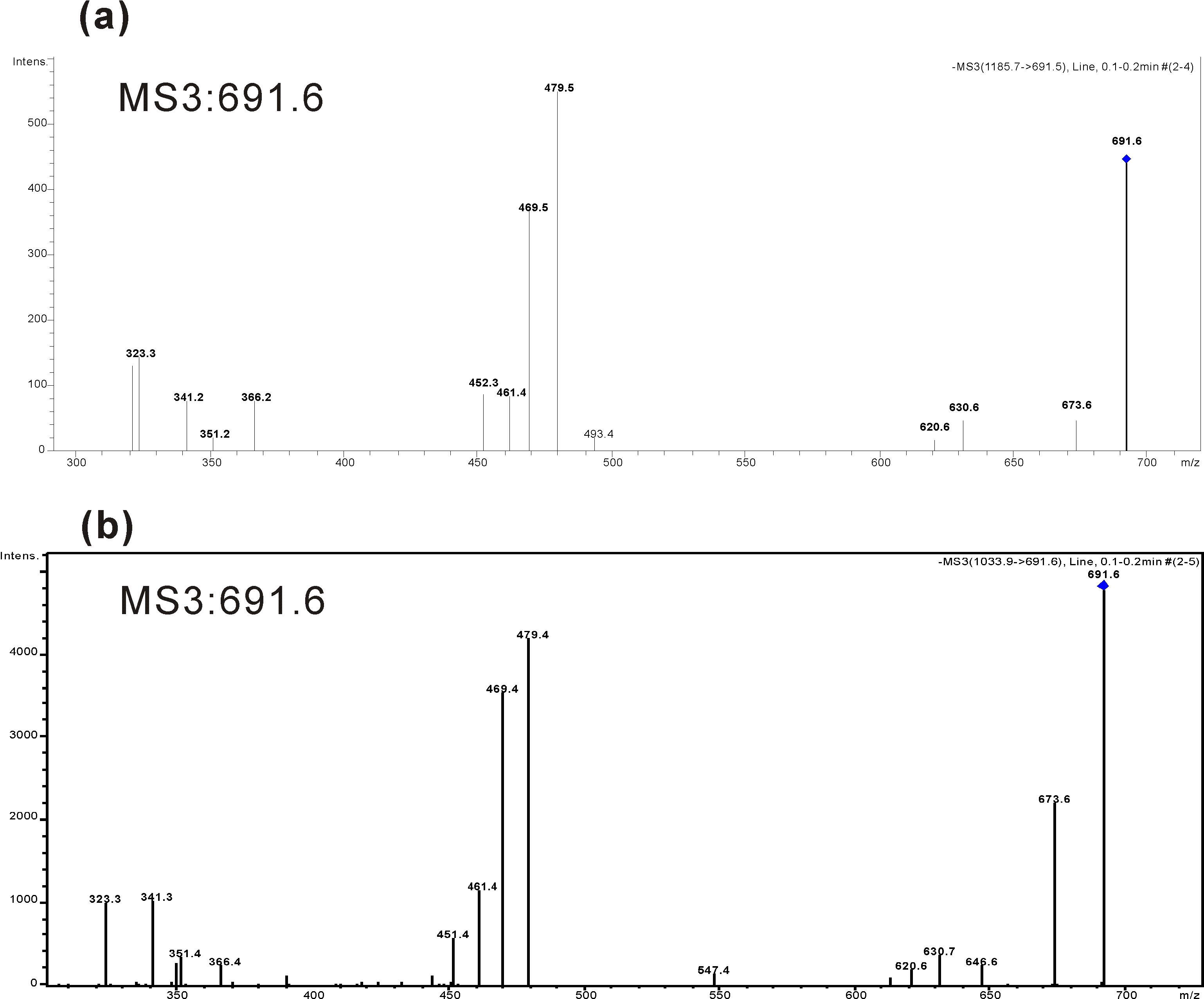
*

Figure S1. The MS^3^ fragmentation of 1016 Da ions of non-covalent complex (a) and lichenysins G (b).


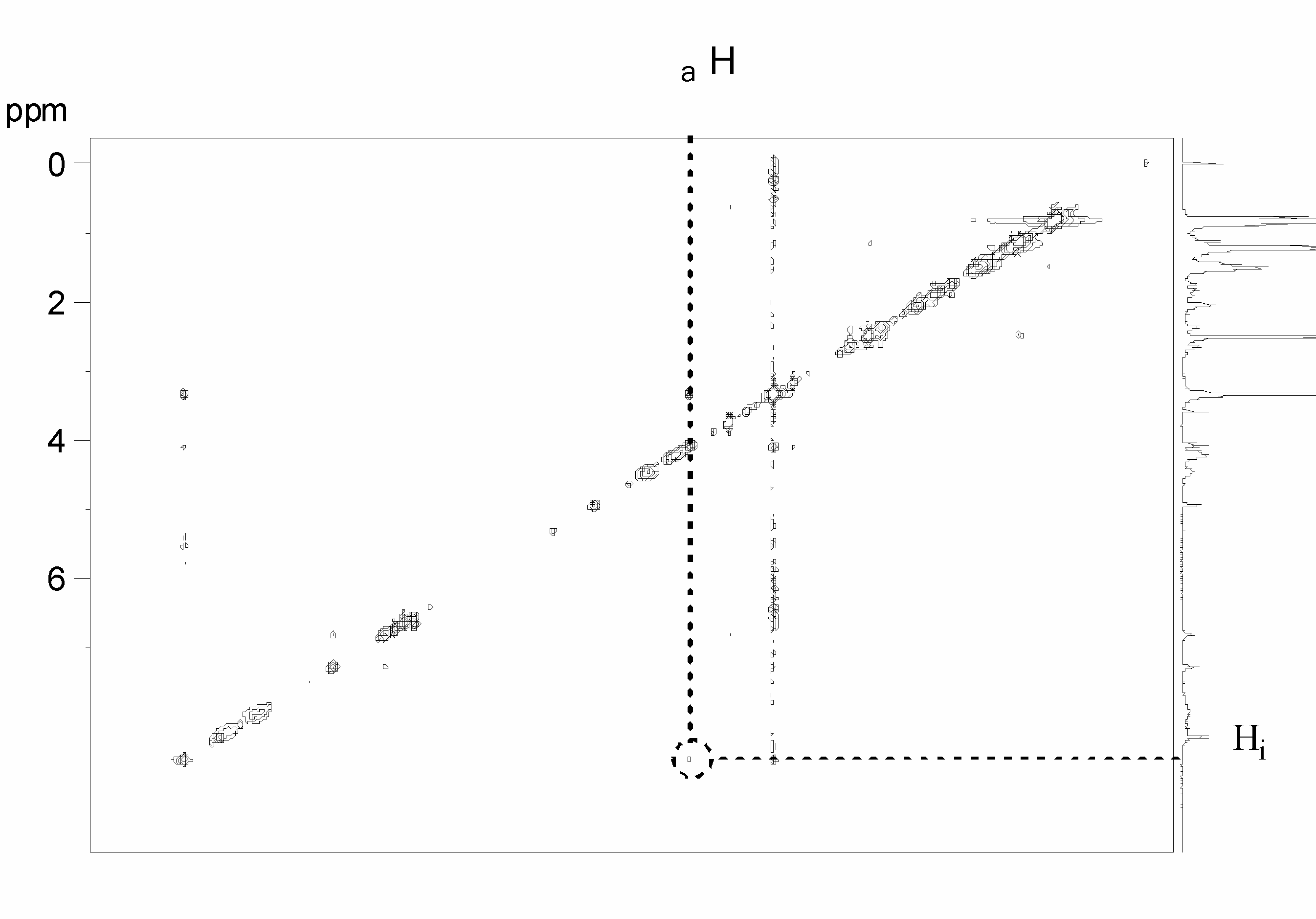


Figure S2. ^1^H-^1^H NOESY spectra (400MHz) of non-covalent complex (1:1 in molar ration )in DMSO.

Table S1, The calculation of non-covalent complex and lichenysins G with ONIOM level of theory

| NAME | Energy | Relative energy | Frequency |
| --- | --- | --- | --- |
| Reactant1-1 | -724.9247710 | 0 |  |
| TS1-1 | -724.7947070 | 81.61639 | -1697.74 |
| Product1-1 | -724.8454090 | 49.80040 |  |
|  |  |  |  |
| Reactant1-2 | -724.8790410 | 0 |  |
| TS1-2 | -724.7896410 | 56.09934 | -624.88 |
| Product1-2 | -724.8639050 | 9.497983 |  |
|  |  |  |  |
| Reactant2-2 | -1225.699786 | 0 |  |
| TS2-2 | -1225.577468 | 76.75571 | -1697.74 |
| Product2-2 | -1225.624833 | 47.03372 |  |
